# Cognitive functioning of patients with borderline personality disorder

**DOI:** 10.1192/j.eurpsy.2024.371

**Published:** 2024-08-27

**Authors:** E. Chumakov, D. Charnaia, N. Petrova

**Affiliations:** Department of Psychiatry and Addiction, Saint Petersburg State University, Saint Petersburg, Russian Federation

## Abstract

**Introduction:**

The neurocognitive deficit model as a characteristic of patients with borderline personality disorder (BPD) has been the focus of research for the past 20 years. However, no such studies have been performed in Russia.

**Objectives:**

The aim of the present study was to investigate the neurocognitive profile of patients with BPD.

**Methods:**

Fifty patients with BPD (according to DSM-V criteria) in stable mental state (72% women; mean age 22.44±4.32) were examined. BPD symptom severity was assessed using the Borderline Personality Disorder Questionnaire (PBQ-BPD), which was validated in the Russian population (34 points or more indicated a higher probability of BPD diagnosis). The Brief Assessment of Cognition in Schizophrenia (BACS) was used to assess cognitive function (in order to use these data for differential diagnosis with schizophrenia spectrum disorders). The study was approved by the ethical committee of Saint Petersburg State University.

**Results:**

On the PBQ-BPD results, 38% of patients (n=19) scored over 34 points, despite being stable. BACS subscales T-scores (presented as median [Q1; Q3]) were within normal limits (Verbal memory - 49.81 [46.56; 53,06]; Working memory - 43.73 [38.0; 47.50]; Motor function - 44.08 [41.0; 47.25]; Coding - 45.56 [42.50; 48.63]; Verbal fluency - 48.14 [46.0; 52.0]; Tower of London test - 52.33 [47.0; 57.0]). A number of patients had low scores on the BACS subscales (T-score < 40), particularly working memory (33.3%), coding (20.8%), and verbal memory (18.8%). The BACS Composite T Score (46.02 [43.65; 48.39]) correlated with the PBQ-BPD score (32.00 [27.00; 36.00]; r=-0.316; p=0.028). To better characterize the cognitive functioning of patients with BPD, patients were divided into two groups: those who scored less than 34 on the PBQ-BPD (group 1) and those who scored more than 34 on the PBQ-BPD (group 2). Group 2 patients had a lower BACS Composite T-score (42.32 [38.06; 46.58]; 48.45 [45.87; 51.03]; p=0.009) and nominally lower mean scores on all BACS subscales, compared with Group 1 patients. We found significant differences in T-scores values on the Working Memory subscale (Group 1 - 45.0 [41.0; 49.0]; Group 2 - 38.0 [33.0; 43.5], p=0.003), Verbal Fluency (49.0 [47.25; 53.75]; 48.0 [44.0; 49.0]; p=0.047), Tower of London Test (57.0 [52.0; 57.0]; 48.0 [42.0; 57.0]; p=0.036).

**Conclusions:**

Neurocognitive impairment was detected in 33.3% of patients with BPD. The dominant cognitive impairments in the patients were decreased working and verbal memory and information processing speed. The severity of BPD symptoms has been confirmed to correlate with the neurocognitive functioning of these patients.

**Disclosure of Interest:**

None Declared

